# Sustainability in anaesthesia and critical care: beyond carbon

**DOI:** 10.1016/j.bjae.2022.08.005

**Published:** 2022-10-31

**Authors:** L. Fang, R. Hixson, C. Shelton

**Affiliations:** 1 North West School of Anaesthesia, Manchester, UK; 2 County Durham and Darlington NHS Foundation Trust, Darlington, UK; 3 Manchester University NHS Foundation Trust, Manchester, UK; 4 Lancaster Medical School, Lancaster University, Lancaster, UK

**Keywords:** environment, environmental pollution, oceans, public health

**Learning objectives**

By reading this article, you should be able to:

• Outline the importance of the biosphere and its constituent parts.
• Describe the impact of pollution and climate change on each other and on the overall planetary health.
• Detail the impacts of healthcare-related activities on environmental pollution, from procurement to use to waste.
• Adopt strategies to mitigate some of the effects of healthcare on the environment and on health.

**Key points**

• Earth's capacity to support life has reduced in recent decades, driven by pollution, habitat loss and climate change.
• Medical equipment and drugs contribute to environmental harms through the extraction of raw materials, manufacturing, transport and disposal. This is exacerbated by excess waste, including disposable and single-use equipment.
• Drugs and their metabolites (notably propofol and antibiotics) are commonly discharged into water by wastage or excretion. Their effects on aquatic life can be considered in terms of persistence, bioaccumulation and toxicity.
• Efforts to mitigate the environmental impacts of healthcare should be holistic in their approach to risks and benefits; clinicians and policymakers should attempt to balance greenhouse gas emissions with other harmful effects.
• Strategies to address the ecological impacts of healthcare include reducing consumption, avoiding disposable or single-use equipment, recycling and eliminating waste.

Our planet is in the midst of an ecological crisis. This ecological crisis is most often discussed in terms of global warming, described by the Lancet Commission as ‘the biggest global health threat of the 21st century’. Healthcare services are now dealing with an increasing number of climate change-related pathologies, but as a major producer of greenhouse gases (GHGs) they are contributing to their own workload. Carbon dioxide equivalent (CO_2_e) is the measure used to account for emissions from all gases that contribute to global warming, including CO_2_, methane, nitrous oxide and others. Globally, it is estimated that healthcare accounts for 4% of overall carbon emissions; if healthcare were a country, it would have the fifth largest carbon emissions in the world.^1^ There is therefore increasing scrutiny of healthcare-related carbon emissions. The NHS in England, for example, has recently committed to achieving carbon ‘net zero’ by 2040 and demonstrated a reduction in ‘carbon footprint’ of 580 kt CO_2_e in 2021. Around 5% of acute hospitals’ carbon emissions are from inhalational anaesthetic agents, and this has focused anaesthetists’ attention on their role in mitigating the carbon footprint of healthcare.^2^

**Clinical scenario**

Mrs J is a 70-yr-old woman with hypertension and Type 2 diabetes mellitus, who is listed for an elective total knee replacement after consultation with an orthopaedic surgeon. Two weeks before surgery, she attends a preoperative clinic and has blood tests and an ECG. Two days before surgery, she attends hospital for SARS-CoV-2 screening.

On the day of surgery, her husband drives her to hospital. She gets changed into a hospital gown, her observations are taken and an i.v. cannula is inserted. She is seen by her surgical and anaesthetic teams.

Mrs J, who tries to lead a sustainable lifestyle, has noticed that her preoperative care has involved a lot of transport and has already generated a lot of waste. She is apprehensive that her operation may be similarly resource-intensive and asks the doctors about the medicines, equipment and energy involved in surgery (Fig. 1). She wants to know what the hospital is doing to make her care ‘greener’.

**Fig 1:**
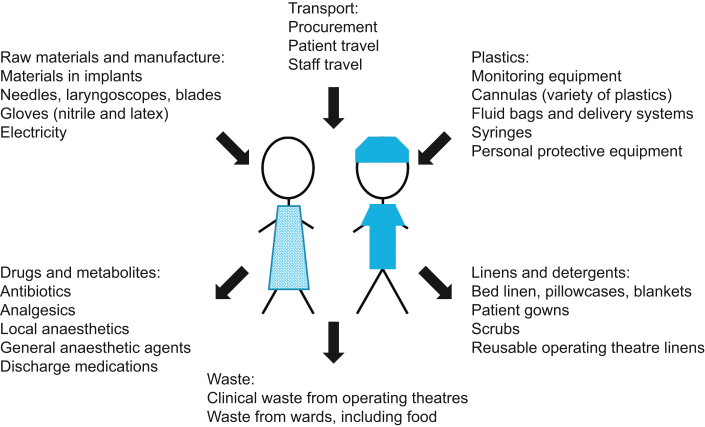
Examples of resource use and disposal in the perioperative period.

Reducing the emission of GHGs is a key tenet of sustainable healthcare practice. However, focusing on carbon at the exclusion of other concerns, such as ecotoxicity, air quality and water pollution, is problematic (Fig. 2), not least because neglecting these factors may contribute to worsening climate change by damaging Earth's defences against global warming. In this article, we move ‘beyond carbon’ by describing the systems that comprise Earth's biosphere, outlining the broader environmental implications of anaesthesia practice on these systems and suggesting ways to mitigate negative impacts on planetary health.

**Fig 2:**
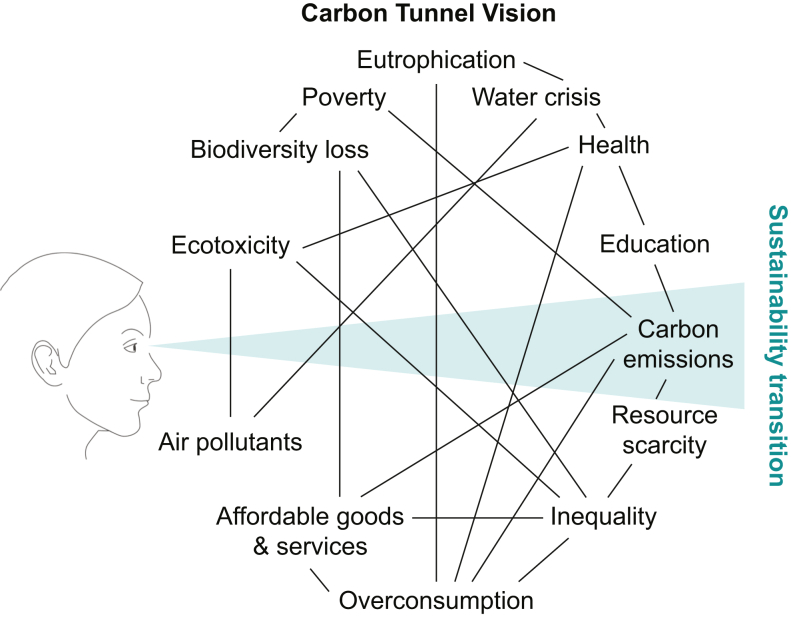
Carbon tunnel vision. Graphic reproduced with permission from the artist, Jan Konietzko.

## The biosphere

The biosphere is where life occurs on Earth. It comprises the lithosphere (land), atmosphere (air) and hydrosphere (water). The components of the biosphere are interconnected and mutually reliant.

### The hydrosphere

Oceans cover 70% of Earth's surface, contain 97% of Earth's water and 80% of all living organisms and produce 50% of Earth's oxygen. They act as a buffer to climate stressors, absorbing 90% of excess heat energy and 25% of carbon dioxide. Oceans store 40 trillion metric tonnes of carbon (16 times more than land and 50 times more than the atmosphere). However, as acidification increases, oceanic capacity to absorb further carbon decreases, thereby reducing buffering against global warming.^3^

Global warming directly affects the health of oceans. Effects include marine heat waves and rising sea levels, which are projected to rise by at least 29 cm by 2100, resulting in a 100-fold increase in the risk of flooding.^4^ Oxygen solubility is reduced in warmer water, with more profound effects in the mid-water region (1,000–5,000 m depth), having an impact on many marine species. Deoxygenation also affects coastal waters, and its effects are amplified by land-derived nutrients causing eutrophication and the multiplication of *Vibrio* bacteria and harmful algal blooms.^4^ These effects are changing ocean currents, impacting on fish populations and migration. Aside from the ecological damage, this impact on fish stocks affects livelihoods and the food supply chain.

**Fig 3:**
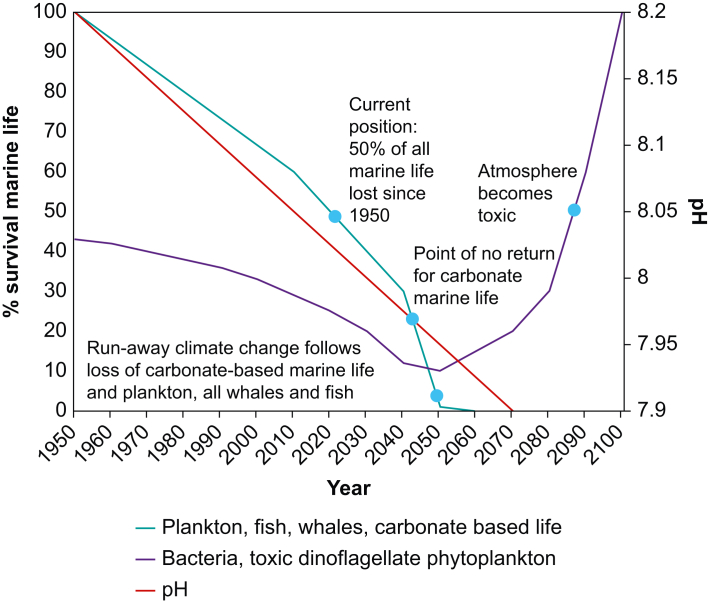
Past and projected carbonate-based marine life survival against ocean pH. Climate-regulating ocean plants and animals are being destroyed by toxic chemicals and plastics, accelerating our path towards ocean acidification. Redrawn from Dryden and Duncan, with the permission of Goes Foundation.^3^

Plankton thrive in acidic environments, yet their biomass has declined by 50% in the last 70 yrs, along with overall marine life.^3^ This gap is thought to be because of toxic chemicals and microplastics, which affect phytoplankton and zooplankton, even in extremely low concentrations. Most of these chemicals originate on land, and over this same period of time the human population has tripled. The pH of oceans is currently around 8.1 and has decreased significantly over the last century. Now, large areas of oceans are devoid of life, and if ocean pH drops to 7.95, an estimated 80–90% of all marine life will be lost (Fig. 3).^3^

The combined effect of climate change and pollution on the oceans is damaging the biological carbon pump that is driven by phytoplankton. The reduced ability of the oceans to mitigate against rising atmospheric CO_2_ results in further acidification and a dangerous positive feedback loop. Climate change efforts, therefore, need to be coupled with action to stop toxic chemicals from reaching the oceans and waterways, which is one of the five ‘tipping points for a healthy and productive ocean’ outlined by the United Nations Global Compact.^5^ If oceans ‘fail’, humanity will simply cease to exist.

### Lithosphere and atmosphere

The decline in capacity of the biosphere to support life is also reflected on land, with studies showing 10% year-on-year decline in insect biomass. The main driver behind this decline is pollution.^3^

Outdoor air pollution is the fifth commonest cause of mortality worldwide, accounting for 4.2 million deaths annually. Major pollutants include sulphur dioxide, nitrogen oxides, volatile organic compounds (VOCs) and particulates.^6^ Emissions can be from natural sources, such as volcanoes, but the majority are human-made, most significantly arising from fossil fuel combustion. Primary air pollutants are those pollutants released directly from the source, and secondary pollutants result from interaction between compounds in the atmosphere (e.g. ozone from nitrogen oxides and VOCs in the presence of sunlight). Air pollution is exacerbated by global warming, as higher temperatures accelerate the formation of some pollutants and increase dispersal.^7^

Deforestation is the second largest human-made source of GHG emissions after fossil fuel combustion. Although not as significant as the oceans, rainforests act as a global carbon sink. The Amazon rainforest alone is estimated to store up to 200 billion imperial tons of carbon but has seen 20% of its area deforested in the last 60 yrs, with another 20% at risk.^8^ The main causes of deforestation include forest clearance for agriculture, most notably for the production of soya beans (the main component of the i.v. formulation of propofol) and palm oil, and droughts and fires caused by climate change itself.^9^

Shoreline erosions can force plant growth further back and expose tree roots, leading to tree fall. This increases the risk of flooding and causes the resuspension of sediments, which impacts aquatic plants and fish spawning; habitat destruction; and, ultimately, biodiversity loss.

## ‘Where things come from’: embodied impacts

### Raw materials and manufacture

Numerous materials are involved in healthcare, with metal and plastic making up the majority of equipment and disposables used in anaesthetic practice. The use of disposable medical equipment has more than doubled between 2005 and 2020, and the increased use of personal protective equipment (PPE) in the COVID-19 pandemic will further exacerbate this trend.^10^

#### Metal and minerals

Mining is an energy-intensive process, accounting for approximately 10% of energy-related GHG emissions worldwide and inevitably results in environmental damage: excavation leads to deforestation and soil erosion, harmful compounds are emitted to air and water, whilst metal and other persistent particles mix with soil in the surrounding area, preventing future plant growth.^11^ Specific issues exist with mining for certain materials; for example, mining for titanium ore is associated with radionuclide release, groundwater pollution and toxicity from chlorine used for cleaning.

Metal manufacturing is associated with further impacts. For example, the production of stainless steel (used in devices such as cannulas, needles and laryngoscope blades) generates pollution from coke production, which emits naphthalene, ammonium compounds and sulphur dust. Stainless steel contains 10–20% chromium, an environmental toxin that impairs plant photosynthesis and is associated with respiratory disease, neurological damage and tumour formation in animals and humans.^12^

Aside from long-lasting impacts on biodiversity and human health, issues exist with the use of conflict minerals in medical equipment. For example, 60% of cobalt, used in surgical implants and batteries, is mined from the Democratic Republic of the Congo, which has ongoing human rights issues in the mining workforce, including child exploitation.^13^

#### Plastic and rubber

Virgin plastic is extracted from petrochemicals, such as natural gas or crude oil. Hydraulic fracturing (fracking) has been increasingly used to meet increasing demand for ethylene and propylene, the most important plastic feedstocks.^14^ There is evidence that fracking wastewater (containing salts, organic matter and radioactive materials) enters the drinking water supply, and an association has been found between proximity to fracking wells and hospitalisation for cardiac and neurological disease.^15^ The US Environmental Protection Agency lists cancer, neurotoxicity, liver and kidney damage and developmental damage in children as some of its effects.^16^ Processing fossil fuels into plastic resins also releases carcinogens and neurotoxic agents.

Disposable gloves account for a significant proportion of single-use medical items; obtaining rubber for latex gloves is a major contributor to deforestation, and nitrile gloves are made from a co-polymer of acrylonitrile and butadiene, derived from petroleum.

### Transport

All modes of commercial transport are associated with the emission of GHGs, such as carbon dioxide, methane and nitrous oxide. Although concomitantly released pollutants, such as sulphur oxides, nitric oxides and particulate matter, have an atmospheric cooling effect, this is outweighed by GHG emissions. Emissions from aircraft cause disproportionately greater climate effects, as they are released high in the atmosphere. Sea freight, which accounts for the greatest proportion of commercial transport, has the lowest GHG emissions but has other important environmental impacts.

#### Sea freight

Sea freight accounted for an estimated trade of 11.08 billion tonnes in 2019. Whilst it has advantages in terms of lower GHG emissions per unit transported, it is responsible for harmful discharges to water.^17^ Oil discharges are part of normal operations, making up >70% of total oil spills. Effects of oil spillage include DNA damage in aquatic life, toxicity to eggs, damage to bird plumage and reduced filtering capacity in baleen whales. Wastewater discharged from ships contains sewage, microbes, pharmaceuticals, detergents and microplastics. Marine litter, most commonly plastics, can cause entanglement and accidental ingestion. Anti-fouling paints applied to ships’ hulls contain biocides to prevent organism accumulation but also inhibit photosynthesis in plankton and corals when they leach into the water. Non-indigenous species can be transferred outside of their natural habitats. This has adverse effects on biodiversity and can spread diseases outside their usual range.

Underwater radiated noise is produced predominantly from ship propellers because of cavitation and turbulent flow, in the 10 Hz to 1 kHz range.^17^ This disrupts communication, navigation, mating and habitat selection for a wide variety of organisms and is thought to cause long-term effects on behaviour and physiology. Because sound travels faster and further in water compared with air, these effects are far reaching. Wildlife collisions are well documented, with many fatal collisions involving endangered species of whales, sharks and sea turtles, all an essential part of the finely balanced oceanic ecosystem.

#### Road transport

Road travel related to the NHS is responsible for 3.5% of all journeys in England, resulting in around 7,285 tonnes of nitric oxide and 330 tonnes of particulate matter (specifically tiny particle of 2.5 microns or less [PM 2.5]).^2^ It is estimated that air pollution leads to 40,000 deaths per year in the UK, so NHS-related travel could be responsible for 1,400 of these deaths.^18^

Around 30% of NHS-related journeys are for business and fleet transport. Although the electrification of NHS fleet vehicles will reduce emissions, pollutants arising from the abrasion of car tyres on road surfaces will persist. Tyres are made of natural and synthetic rubbers, sulphur and zinc, and they produce particulates, including microplastics.^19^ They lose 10–20% of their weight during their lifespan from wear, equating to an average 63,000 tonnes per year of emissions in the UK, with larger particles entering rivers and oceans *via* surface run-off. Smaller particles become airborne, can be inhaled and deposit into the oceans, contributing 5–10% of oceanic microplastics.

## ‘Where things go to’: direct impacts

### Plastics

Most single-use medical equipment is made from, and packaged in, plastics. Globally, medical waste makes up 4% of total plastic waste, and before the COVID-19 pandemic plastics made up 23% of the total waste in the NHS.^20^

Every step in the life cycle of plastics damages environmental and human health through GHG emissions and contamination of air, water and soil. The majority of plastics’ lifespan and impacts occur after their use. In the water, macroplastics are responsible for marine animal deaths, directly through ingestion and entanglement and indirectly by transporting toxins in higher concentrations than the surrounding water. Microplastics are ingested by plankton, affecting their growth and reproduction and limiting their ability to sequester carbon (although the exact mechanisms are under research). Microplastics also enter the human body *via* inhalation and ingestion, and they may increase risks of malignancy.^15^

Despite all this, the plastics industry continues to grow, with the World Economic Forum projecting production and usage to increase by 3.8% per year.

### Drugs and metabolites

Up to 90% of oral medications are excreted into wastewater as active substances (original pharmaceutical form, metabolites or other transformational products) in patients’ faeces and urine.^21^ The majority originate from community prescribing, commonly antibiotics and antidepressants, whilst hospital wastewater also includes specialist agents. Wastewater treatment plants are not designed to remove pharmaceuticals, so some will be discharged into surface water and in sludge used as agricultural fertiliser with low concentrations detected in drinking water.^22^

The Swedish Chemicals Agency classification system for aquatic pollutants can be used to assess the impacts of pharmaceutical waste on the aquatic environment. It comprises

(i) Persistence: ability to resist degradation
(ii) Bioaccumulation: build up in adipose tissue of aquatic creatures
(iii) Toxicity: potential for harm

Table 1 includes the details of selected drugs relevant to anaesthesia and critical care. Little, or incomplete, information is available for some drugs, but pharmacological principles can rationally be applied. For example, cisatracurium can be assumed to have low persistence and bioaccumulation because of Hoffman degradation.

**Table 1:** Persistence, bioaccumulation and toxicity of drugs used in anaesthesia and intensive care.

Drugs	Persistence	Bioaccumulation	Toxicity	Additional notes
Propofol	High; also accumulates on land in biosolid waste	Potential; because of high lipid solubility	Multiple effects, including algae growth inhibition and acute toxicity in small crustaceans and freshwater fish	Falås and colleagues showed higher levels of propofol *leaving* wastewater treatment plants than *entering*, possibly because of deglucuronidation.^23^ As 60 times more propofol is excreted in the glucuronidated form, currently measured levels are likely hugely underestimating the environmental risk.

Opioids	High	Potential	Cause genetic damage to bivalves and water fleas	Few studies into remifentanil but has low persistence and bioaccumulation and likely low toxicity

Antibiotics	Macrolides and quinolones: high; overall, antibiotics exhibit pseudo-persistence	Some have high bioaccumulation.	Multiple antibiotics with toxicity to fish and amphibians; beta-lactams generally less toxic	Macrolides are toxic to zebrafish, affecting yolk sac and swim bladder. Tetracyclines toxic to amphibians.

Lidocaine	Monoethylglycinexylidide (MEGX), a major metabolite, is highly stable at room temperature	Low because of low lipid solubility	Possible; MEGX metabolises to 2,6-dimethylaniline, a likely carcinogen	∼10% of lidocaine given is excreted unchanged in the urine

Bupivacaine	High	Little evidence, but likely low at pH 7.4	Low; little data on ecotoxicity of metabolites	Metabolised in liver, excreted in urine and <10% unchanged.

Sugammadex	Lack of literature currently	Lack of literature currently	Binds to oestrogen and progesterone, so concern with endocrine disruptor for aquatic life	Eliminated unchanged by the kidneys.

Paracetamol	<15 days in the environment, but pseudo-persistence	Effluent release areas constantly exposed, leading to chronic exposure	Highly toxic to bacteria and algae; neurotoxic to crustaceans and planarians at low doses	Some degradation products may be more toxic than paracetamol itself, adding to the total environmental impact.

Propofol, one of the most commonly used drugs we use, is also one of the most commonly wasted, with 33–50% of drawn-up propofol being discarded.^24^ After being given to the patient, <1% is excreted unchanged and 60% undergoes hepatic glucuronidation.^25^ Unsafe disposal into sinks or general waste results in soil and water pollution, where propofol has a half-life of >1 yr.

Antibiotics cause direct ecological effects as a contaminant and indirect health effects through antibiotic resistance. In water, they affect fish by disrupting gut microbiota, inhibiting growth and causing infertility.^26^ On land, they affect earthworm enzyme activity and plant protein synthesis, and they reduce the activity of soil microbes, limiting the availability of nitrogen and CO_2_ required by plant life. Whilst beta-lactams are less toxic, they affect chloroplast division in lower plants (e.g. algae and mosses) and photosynthesis. Antimicrobial resistance causes more than 33,000 deaths and the loss of more than 874,000 disability-adjusted life years in Europe each year.^27^ This is projected to worsen with antibiotic-resistant genes increasingly found in environmental samples. Incorrect disposal of antibiotics *via* wastewater is of particular concern, as sewage is rich in nutrients with high concentrations of bacteria, so is a hotspot for the development of resistant strains.

### Linens and detergents

Laundry detergents contain not only soap but a multitude of chemicals (surfactants, sequestration agents, enzymes and bleaching agents) that all contribute to environmental damage. The wastewater produced may also contain organic matter, oil and microplastics.

Surfactants (some of which are made from palm oil derivatives) disrupt the natural oils in fish, damaging gills and increasing vulnerability to other toxins. Whilst most surfactants are successfully treated in sewage treatment plants, some remain as persistent organic pollutants.

Sequestrant agents increase the effectiveness of detergents. Phosphate was banned in the European Union because its fertilising effect in water caused algal blooms, deoxygenation and fish death. Current alternatives include ethylenediamine tetra-acetic acid (EDTA), which has low toxicity and moderate persistence, but increases the bioavailability of heavy metals, therefore indirectly causing toxicity.^28^

Bleaching agents, which cause oxidation of coloured molecules, include chemicals, such as hydrogen peroxide, which are directly biocidal.

### Waste

The NHS generated 624,000 tonnes of waste between April 2019 and March 2020. Of this, 47% is incinerated, 16% is recycled, 7% goes directly to landfill, with the remainder undergoing alternative treatments.^29^ Since the COVID-19 pandemic, the amount of medical waste has been found to have increased by up to 350% in some countries, mostly from plastic PPE.^30^

#### Incineration

Incineration is used for waste disposal and generation of electricity. However, waste is incompletely destroyed, with ash, wastewater, sludge, air pollutants and combustion gases remaining after incineration. Fly ash (carried by smoke) and bottom ash (which falls to the bottom of the combustion chamber) compose 30–50% of the original waste volume and are usually then transported to landfill. This means that approximately one-third of NHS waste ultimately ends up in landfill.

Fly ash is readily windborne, and although commercial incinerators take measures to capture it, this process is incomplete. Alongside the known harmful substances found in ash (Table 2), multiple compounds have unknown potential for harm. Studies have shown higher rates of cancer and birth defects around municipal waste incinerators.^31^ As they are usually located in socioeconomically deprived areas, this also contributes to worsening health inequalities.

**Table 2:** Examples of common hazardous materials related to waste disposal. COPD, chronic obstructive pulmonary disease; CVS, cardiovascular system; GI, gastrointestinal; KUS, kidney and urinary system; Misc, miscellaneous; NS, nervous system; RS, respiratory system.^31^

Chemical component	Background	Acute effects	Chronic effects
Cadmium *Route: inhalation and ingestion*	Highly toxic to animals and humans at low concentrations. Acute poisoning is uncommon because of monitoring.	RS: breathing difficulty, cough with bloody sputum, pneumonitis and pulmonary oedema GI: nausea and vomiting and abdominal cramps NS: irritability, weakness, convulsions and coma	RS: bronchitis and emphysema KUS: renal failure GI: nausea and liver failure Misc: osteomalacia, anaemia and pain Carcinogen *via* inhalational route

Chromium *Route: inhalation, ingestion and dermal*	Hexavalent chromium is efficiently absorbed and highly toxic; trivalent is generally insoluble so less toxic.	RS: wheeze, cough and chest pain Misc: ulceration of nasal septum, laryngitis and allergic reaction	RS: lung cancer

Lead *Route: inhalation and ingestion*	Waste disposal contributes 7% of airborne lead in UK. Acute poisoning is rare; children <6 yrs are most at risk.	GI: pain, nausea, vomiting and diarrhoea NS: headache, weakness, numbness and tingling	CVS: hypertension KUS: renal failure GI: pain and cramps NS: learning difficulties, autism and irritability Misc: anaemia and reduced fertility

Dioxins (polychlorinated dibenzodioxins) *Route: dermal and ingestion*	Low volatility and water solubility, but high lipid solubility and long half-life. Air emission from incineration; adsorbs to particles and soil	Typically dermal entry GI: nausea, vomiting and liver fibrosis NS: headache Misc: chloracne, muscle pain and weight loss	Typically ingestion *via* accumulation in food chain NS: developmental delays Misc: soft tissue sarcomas and endocrine disruption

Polycyclic aromatic hydrocarbons *Route: inhalation, ingestion and dermal*	Carbon and hydrogen, with two or more fixed benzene rings. Produced by high-temperature reactions; highly persistent.	Low risk generally RS: respiratory tract irritation Misc: eye irritation	Known carcinogens RS: eight-fold increase in risk of lung cancer KUS: bladder cancer Misc: skin cancer

BTEX compounds (benzene, toluene, ethylbenzene and xylenes) *Route: inhalation and ingestion*	Biodegradable under anaerobic conditions. Half-life of a few days in air, but water soluble.	Benzene is most toxic. NS: coma and death Misc: neutropaenia	Known carcinogens Misc: leukaemia, foetal toxicity and abortion

Particulate matter *Route: inhalation*	Smaller particulate matter have greater effect (especially PM 2.5). Adsorb carcinogens from incineration and carry them into human body.	RS: cough, wheeze and acute exacerbation of asthma	RS: asthma, COPD and lung cancer CVS: blood clots and increased mortality from ischaemic heart disease Misc: general inflammatory effects

Sulphur dioxide *Route: inhalation and dermal*	Colourless gas with pungent smell. Water soluble; forms sulphuric acid. Main other source from industry and motor vehicles.	RS: cough, mucus secretion and acute exacerbation of asthma Misc: skin damage	RS: asthma CVS: worsens existing disease and increases risk of death

Nitrogen oxides (nitrogen oxide and nitrogen dioxide) *Route: inhalation*	Produced by combustion process, mainly motor vehicles.	RS: bronchoconstriction even in people without asthma	RS: decreased lung function in children and worsening of asthma and COPD

#### Recycling

Plastics do not decompose or biodegrade—they remain plastics forever, only becoming smaller. Unlike some materials (e.g. paper, glass and aluminium), plastics degrade in quality with every cycle, which limits regeneration of medical equipment through recycling. ‘Downcycling’ of medical plastics (refabricating into other lower-quality products) merely postpones the eventual disposal of the plastic. Nevertheless, recycling plastics is three times more efficient than incineration in terms of GHG emissions, but recycling of plastics used in clinical practice can be hampered by issues with sterilisation and sorting.^14^

Paper recycling is preferable to both production of new paper and disposal in landfill. Recycling is associated with lower emissions of particulate matter and sulphur dioxide to air and water pollution (e.g. perfluorinated alkyl substances) than pulp production, and lower energy requirement to process.^32^ It is also associated with lower GHG emission compared with landfill, as paper biodegrades into methane and CO_2_.

#### Landfill

Landfill sites are constructed with bottom liners, leachate collection systems and a final cover, with modern sites designed to minimise emissions. Regulations are in place to monitor pollution levels, but these systems are not fail-proof, and further research on landfill emissions is still required. Further ecological impacts include landscape changes and loss of vegetation and habitats. Socioeconomic impacts include public health risks and litter in the surrounding environment. As with incinerators, landfill sites tend to be in more deprived areas.

The major pollutants emitted from landfill are methane and CO_2_ from biodegradation. Leachate contains a large variety of chemicals, including nitrogen nutrients, toxic organic compounds, heavy metals and VOCs. Flaws or damage to the liners can lead to contamination of the surrounding land and water.^33^

Bioaerosols (pathogens and allergens suspended in the air) have been found at elevated levels near landfill sites. These bioaerosols include fungi, moulds, Enterobacteriaceae and endotoxins, which may cause upper airway symptoms and exacerbation of asthma and, in high-enough levels, infection.^34^

Interestingly, disposing of plastic to landfill has a lower carbon footprint than incineration because plastics resist biodegradation. This demonstrates the importance of a holistic ecological approach, not just focusing on carbon emissions.

## Reducing the ecological impacts of healthcare

Efforts to mitigate the environmental impacts of healthcare should be holistic in their approach to global risks and benefits, and clinicians and policymakers should be wary of the unintended consequences of interventions. Balancing ecological, social and financial impacts (known as the ‘triple bottom line’) on a global scale is profoundly challenging, but the first steps to achieving this involve awareness of the scope of that challenge and implementing strategies that have benefits in all three domains.

In cases like that of Mrs J in the clinical scenario, there are many opportunities to reduce the overall environmental effects of her care. Optimisation of pre-existing conditions and applying ‘getting it right first time’ principles could reduce complication rates, reduce on-the-day cancellations and shorten any inpatient stay. This would save on avoidable activities, waste generation and cost. Implementing leaner patient pathways, such as carrying out all preoperative assessments and investigations on the same day, reduces unnecessary travel and improves efficiency. Functional digital care systems allow the hospital to receive accurate primary care information and screening results obtained elsewhere, which minimises paperwork and resources used in repeating tests. In terms of her intraoperative care, anaesthesia providers should consider ways to minimise waste, for example through prediction of drug requirements and avoiding the unnecessary use of equipment and disposables. Sustainable procurement (i.e. considering environmental impacts as a core element of the procurement process), for example of equipment, implants, devices and disposables, offers a further opportunity to reduce the environmental impacts of healthcare.

Table 3 includes some strategies that have been found to be effective for environmental effects besides carbon emissions and proposes some areas of future research. All strategies must be supported by improving healthcare staff literacy in planetary health, and anaesthetists can be at the forefront of enabling this education.^35^

**Table 3:** Strategies for reducing the ecological impacts of healthcare and future research opportunities.

Reduce demand for healthcare services and pharmaceuticals:
- Promotion of health, well-being and active lifestyle
- Preoperative optimisation of health to reduce risk of complications and need for resources
- Adhere to ‘green’ formularies (e.g. the Wise List in Region Stockholm)
- Stewardship activities to curb inappropriate prescribing
- Promote practices and pharmaceuticals that are less polluting

Promote ‘green’ and ‘blue’ procurement:
- Assurance from suppliers that goods are manufactured sustainably and ethically, including pharmaceutical companies regarding the origins of soya bean oil used in propofol
- Ensure cargo owners ship goods using the least environmentally harmful methods considering both emissions and impacts on ecosystems
- Ensure all NHS suppliers fulfil the contractual requirement of Policy Procurement Note 6/20 to support environmental protection and improvement
- Quantify the impact on natural capital from the manufacture of shipping of healthcare goods ensuring this impact is considered during the procurement process
- UK distribution networks must reduce impact from emissions and other particulates, such as tyre degradation

Reduce use of consumables:
- Maximise reuse and repurposing of equipment and materials
- Use propofol 2% for TIVA of long duration
- Reduce unnecessary glove use (such as GOSHs Gloves Off Campaign)
- Liaise with manufacturers to remove redundant items from procedural packs
- Reduce reliance on single-use items; use only when reuse and repurposing are not possible

Reduce the impact of waste and disposal (especially plastics):
- Maximise recycling opportunities
- Reusable sharp bins and paper-based containers for non-sharp operating theatre and medication waste
- Improvement of incineration monitoring and safety
- Follow protocols to safely dispose of pharmaceutical waste
- Implementation of sustainable laundry practices for reusable textiles
- Filtration systems for hospital wastewater to prevent pharmaceutical discharge

Further research:
- 2021 to 2030 is the United Nations Decade of Ocean Science for Sustainable Development, aiming to achieve greater cooperation and shared understanding of science to deliver key societal outcomes, including a clean and resilient ocean.
- Develop alternatives to plastics to reduce reliance on petro-chemicals.
- Feasibility of urine collection systems following administration of certain pharmaceuticals (e.g. ongoing trial in Germany involving urine collection bags for patients in the first 24 h following i.v. contrast)
- Reassess and rationalise infection control measures to reflect the latest science and taking into account ecological concerns

## Conclusions

We should extend the principle of ‘first do no harm’ from the patient in front of us to the planet and its citizens. In particular, oceanic health is in decline, with pH forecast to drop to 7.95 by 2045 as the population of phytoplankton, perhaps our greatest ally in fighting climate change, continues to decrease because of pollution. Global inequalities and habitat loss contribute to zoonotic diseases and the risk of further pandemics, which would increase healthcare activities and their already significant contribution on the environment and humanitarian inequalities. These factors feed into a feedback loop that continually worsens the state of the planet and conditions for the living organisms on it.

It is clear that carbon net zero does not equate to environmental net zero. Anaesthetists encounter more than volatile gases in their work, and the procurement, use and waste of drugs and disposables cause ecological damage alongside GHG emissions. Whilst some processes have advantages over others (e.g. recycling compared with manufacture from virgin materials), they all have a negative effect, so the best way to reduce harm to the planet is by reducing consumption. This means everything from rationalising the use of drugs and equipment (such as gloves), avoiding interventions of unclear benefit and choosing less environmentally damaging interventions where there is little difference in clinical effectiveness. This also means focusing on public health and health promotion to lessen the population's need for medical care, so that the negative cycle can be broken.

## MCQs

The associated MCQs (to support CME/CPD activity) are accessible at www.bjaed.org/cme/home for subscribers to *BJA Education*.

## Declaration of interests

CS is a former trainee editor of *BJA Education*. RH is the cofounder of Healthcare Ocean. LF is currently seconded to NHS England as a Chief Sustainability Officer's clinical fellow.
